# Treatment of poorly differentiated neuroendocrine carcinomas of rectum and anus with chemoradiotherapy: a single-centre evaluation

**DOI:** 10.1007/s00432-024-05635-3

**Published:** 2024-03-06

**Authors:** Louise Elkjær Fløe, Ninna Aggerholm-Pedersen, Elizaveta Mitkina Tabaksblat

**Affiliations:** 1 Department of Oncology, Aarhus University Hospital, Aarhus, Denmark; 2 Department of Oncology, EURACAN, Aarhus University Hospital, Aarhus, Denmark; 3 Department of Oncology, ENETS Centers of Excellence, Aarhus University Hospital, Aarhus, Denmark

**Keywords:** Neuroendocrine carcinomas, Neoplasm, PDNEC, Neoadjuvant chemoradiotherapy

## Abstract

**Purpose:**

Poorly differentiated neuroendocrine carcinoma (PDNEC) of the rectum and anus is a rare disease exhibiting aggressive biological behaviour, even if diagnosed early. Currently, there are no agreed standard treatment approaches and management of locally advanced (LA) and metastatic PDNEC usually follows treatments used in pulmonary neuroendocrine carcinomas because of the similarities with small cell lung cancer. The role of surgery in PDNEC is still debated and the benefit of chemoradiotherapy (CRT) is unknown. This report summarises the experiences of CRT application in anorectal PDNEC in a single Danish institution.

**Methods:**

All patients with PDNEC treated with concomitant CRT between May 2019 and January 2021 at a University hospital in Denmark were evaluated. Demographics, treatment and survival outcomes were collected and analysed.

**Results:**

Six patients were identified. Five patients received radiotherapy with 50.4 Gy/28 fractions, and four were eligible for curative resection after the CRT. Distant metastasis was observed in four patients at diagnosis. Two patients with synchronous liver metastases were treated with RFA, and one received a liver resection. The treatment was well tolerated with limited side effects. The median follow-up time was 17 months (range 10–36 months), and the median duration of response was 11.2 months (range 8.1 to 24.2 months). One patient achieved a complete response.

**Conclusion:**

A multimodal treatment approach with CRT in advanced stages of PDNEC in a highly selected patient group is well tolerated and with a high chance of achieving local control and, combined with surgery, even complete response in a single case.

## Introduction

Poorly differentiated neuroendocrine carcinomas (PDNECs) of the rectum and anus are very rare; they comprise about 1–2% of neuroendocrine neoplasms (NEN), but their incidence has risen over the past decades (Dasari et al. 2018; Nagtegaal et al. 2019) Even if diagnosed early, they exhibit very aggressive biological behaviour with short-termed responses to therapy and inferior prognosis compared with common subtypes of rectum and anus cancer (Nagtegaal et al. 2019; Smith et al. 2014; Shafqat et al. 2015). Currently, the treatment of anorectal PDNEC is not standardised. Because of their genetic, pathological and clinical similarities with small cell lung cancer (SCLC), management of metastatic PDNEC usually follows treatment principles used in pulmonary NEC (Shafqat et al. 2015; Janson et al. 2021).

The role of surgery in these neoplasms remains controversial (Janson et al. 2021; Holmager et al. 2022). While recent studies have shown that surgery of the primary tumours in high-grade digestive NEN with the localised or locoregional disease should be considered, both disease-free and overall survival (OS) remain poor (Pommergaard et al. 2021; Sorbye et al. 2013; Merola et al. 2020). Due to the metastatic potential of the PDNEC, postoperative adjuvant platin-based chemotherapy is recommended by most international guidelines (Janson et al. 2021; Garcia-Carbonero et al. 2016; Fields et al. 2019; Chen et al. 2021). However, while the survival benefit of neoadjuvant chemoradiotherapy (CRT) in rectal adenocarcinomas is well recognised, no high-quality data exist supporting the use of perioperative CRT in anorectal PDNEC. Recently published studies have shown an increasing trend towards using CRT in anorectal PDNEC (Della Torre et al. 2021; Antelo et al. 2020; Ueberroth et al. 2021). However, little is known about the optimal treatment schedules, choice of chemotherapy regimens, sequencing of modalities and the appropriate doses of radiotherapy (RT) or CRT. Furthermore, multimodal treatment strategies such as surgery combined with various liver-directed ablative techniques or RT have significantly improved the outcome in advanced rectal adenocarcinomas (Joharatnam-Hogan et al. 2020). Whether these approaches can be extrapolated to managing metastatic anorectal PDNEC is unclear.

This paper aims to report our experience treating patients with anorectal PDNEC with CRT alone, as well as a part of a multimodal approach.

## Material and methods

All patients diagnosed with PDNEC or mixed neuroendocrine–non-neuroendocrine neoplasm (MiNEN) of the rectum and anus treated with RT or CRT at the Department of Oncology at ENETS Neuroendocrine Tumor Center of Excellence, Aarhus University Hospital, from May 2019 to December 2020, were included in this study; the study period ended April 1st 2023, to ensure a proper follow-up time for all patients. This resulted in a cohort of six patients. All patients were initially diagnosed according to fast-track diagnostic multidisciplinary pathways for patients with specific alarm symptoms indicative of colon and anorectal cancer. There were all but one evaluated with MR, CT and PET scans before diagnosis, and the treatment plan was decided at a multidisciplinary team conference (MDT), where oncologists, surgeons, radiologists and pathologists participated. An experienced pathologist confirmed the histopathological diagnosis. Morphological patterns of differentiation, positivity for typical neuroendocrine markers such as synaptophysin and/or chromogranin A, and the proliferative ki67-index assessment were all minimum requirements for the diagnosis.

### Treatment

All patients were treated with one cycle of platin-based induction chemotherapy before beginning radiotherapy. The chemotherapy consisted of either 75 mg cisplatin per m^2^ body surface area (BSA) or carboplatin the area under the curve (AUC) 5 intravenously (i.v.) on day 1. Afterwards, they received 120 mg etoposide per m^2^ BSA, i.v. daily on days 1–3. Cisplatin was offered to patients with no comorbidity, good performance status and a normal glomerular filtration rate. The induction chemotherapy was followed by concurrent CRT, which started on day 22 of the treatment schedule. The patients received 5.5 cycles of chemotherapy on average and only one patient received adjuvant chemotherapy.

Four patients received a total dose of 50.4 Gy in 28 fractions over 5 weeks, and two underwent a short course treatment with 25 Gy delivered in 5 fractions in 1 week.

Gross tumour volumes (GTV) of the primary tumour and involved regional lymph nodes were delineated by the radiologist. Delineation of clinical tumour volume (CTV) and organ at risk (OARs) were adopted from national radiotherapy guidelines for anorectal cancers and tissue Consensus Contouring Guidelines (RTOG). A margin of 5 mm was applied to create a planning target volume (PTV) to ensure optimal coverage. The PTV coverage of at least 95% was achieved for all plans through a volumetric modulated arc therapy (VMAT) dose delivery technique.

The high dose area did not include the small intestine. To attain a good RT plan quality, accounting for interfraction anatomical changes of tumour and OARs volumes as well as achieving target coverage, a systematic monitoring with daily high-quality cone-beam computed tomography (CBCT) was performed. The bowel cavity constraints for the long-course treatment were *V*_30Gy_ < 600 cm^3^ and *V*_45Gy_ < 300 cm^3^, while for the short course, the constraints were *V*_18.5Gy_ < 350 cm^3^ and V_22Gy_ < 200 cm^3^. Acute toxicities were monitored in an outpatient clinic every week and at the end of the treatment.

In all patients, the results of the CRT, feasibility of curative resection of the primary tumour and liver-directed treatment options were discussed at the MDT to ensure an accurate evaluation.

Liver-directed treatments included resection of lever metastases and radiofrequency ablation (RFA) techniques.

The imaging workup before the MDT included a chest/abdomen/pelvic CT scan, an MRI of the lever/pelvic and an FDG PET scan.

After primary treatment, the patients were evaluated with CT scans every 3rd month. The RECIST 1.1 objective assessment criteria for clinical efficacy and tumour response were applied in this study. The Objective Response Rate (ORR) was defined as the percentage of patients who achieved Complete Response (CR) or Partial Response (PR) during treatment.

Common Terminology Criteria graded treatment-related adverse events. For Adverse Events, v4.0, only grade 3 or 4 adverse events were reported.

At progression, the patients received very different treatments raining from palliative radiation therapy, reinduction with platin-based chemotherapy and etoposide, topotecan or best supportive care.

### Data analysis statistics and ethics

Descriptive data are presented, and a swimmer’s plot illustrates patient treatment and follow-up. A local–regional tumour control (LRTC) was defined as the absence of locoregional failure at the time of the analysis.

All statistical analyses were performed by using Stata version 17 (Denmark).

Clinical data were obtained from the patient’s electronic record. All data storage and access complied with the General Data Protection Regulations approved by the Danish patient Safety Authority (no. 3-3013-3276/1). For publication, oral and written informed consent was obtained from patient #6.

## Results

### Patient and tumour characteristics

The median age was 62 years (48–79), and the sex distributions were equal; all the patients were in good performance status at the beginning of treatment. Four of the six patients had distant metastasis at the time of diagnosis, and two were locally advanced. The liver was the most common site of metastasis; one patient had manifestation with limited dissemination to the peritoneum.

The final pathological report confirmed large cell neuroendocrine carcinoma (NEC) in two patients and small cell carcinoma in one patient. The mixed adenocarcinoma and NEC components were present in three patients. The mean ki-67 index was 90% (range 60–100%).

For the detailed patient, tumour and treatment characteristics at the time of diagnosis, see Table 1.

**Table 1:** Patient characteristics, pathology and treatment

*Patient characteristic*	
Patients included	6
Gender (female %)	3 (50)
Mean age (range)	62 (48–79)
PS 0–1 (%)	6 (100)
Mean Ki67% (range)	90% (60–100)
*Morphology*	
Non-small cell/large cell carcinoma (%)	2 (33)
Small cell carcinoma (%)	1(17)
MiNEN^a^ (%)	3 (50)
*Clinical stage at diagnosis*	
Local advanced (%)	2 (33)
Metastatic (%)	4 (67)
Liver metastasis (%)	3 (50)
Peritoneal carcinomatosis (%)	1 (17)
*Chemotherapy*	
Cisplatin/etoposide (%)	4 (67)
Mean cycles	5.5
*Radiotherapy*	
Neoadjuvant (%)	5 (83)
Definitive (%)	1 (17)
Dose 25 Gy/5f (%)	2 (33)
Dose 50,4 Gy/28f (%)	4 (63)
*Surgery of primary tumour*	
Yes (%)	4 (67)
No (%)	2 (33)
R0 resection (%)	3 (73)
R1 resection (%)	1 (25)
*Local treatment for liver metastases*	
RFA^b^ of liver metastasis (%)	2 (67)
Resection of liver metastasis (%)	1 (33)

^a^Mixed adenocarcinoma and PCNEC

^b^Radiofrequency ablation

### Toxicity to treatment

The treatment was generally well tolerated, and no patients had to end the therapy due to side effects. Only one patient underwent a dose reduction in chemotherapy due to fatigue and suppressed haematology. The side effects from RT included pain and skin toxicity, but no grade 3 or 4 severe side effects were observed.

### Efficacy

In total, four patients were eligible to receive curative resection after the CTR, and all patients were eligible for response evaluation. Of the remaining two patients, one was non-operable due to severe comorbidities, and one had systemic progression short after the end of CRT and did not proceed to surgery. Two patients with synchronous liver metastases were treated with RFA (one before CTR and one after), and one received a liver resection.

The relief of disease-related symptoms after one cycle of induction chemotherapy followed by concurrent CRT was observed in the majority of patients.

The median follow-up time after starting treatment was 17 months (range 10–36 months), and one patient (patient #6) achieved a complete response (CR).

A confirmed ORR of 67% was achieved. A significant (38–47%) primary tumour size shrinkage was observed in four patients. The leading cause of treatment failure was distant metastasis. At the time of the disease progression four patients showed no evidence of CT-verified locoregional failure. The distant metastasis had widespread locations, including the brain, liver, lymph nodes and peritoneum. The median duration of response was 11.2 months, ranging from 8.1 to 24.2 months.

The primary tumour size reduction during CRT mostly led to relief of pain, rectal bleeding, tenesmus or alternating stool pattern symptoms.

Pathological reports of postoperative specimens had indicated R0 resection margins in tree out of four operated patients. Furthermore, a decrease in the ki-67 index levels was also observed in some instances.

Pathological reports of postoperative specimens indicated complete pathological response (pCR) on the T-site (ypT0) in two and ypR0 resection margins in three out of four operated patients.

### Patient with complete response

**Fig. 1:**
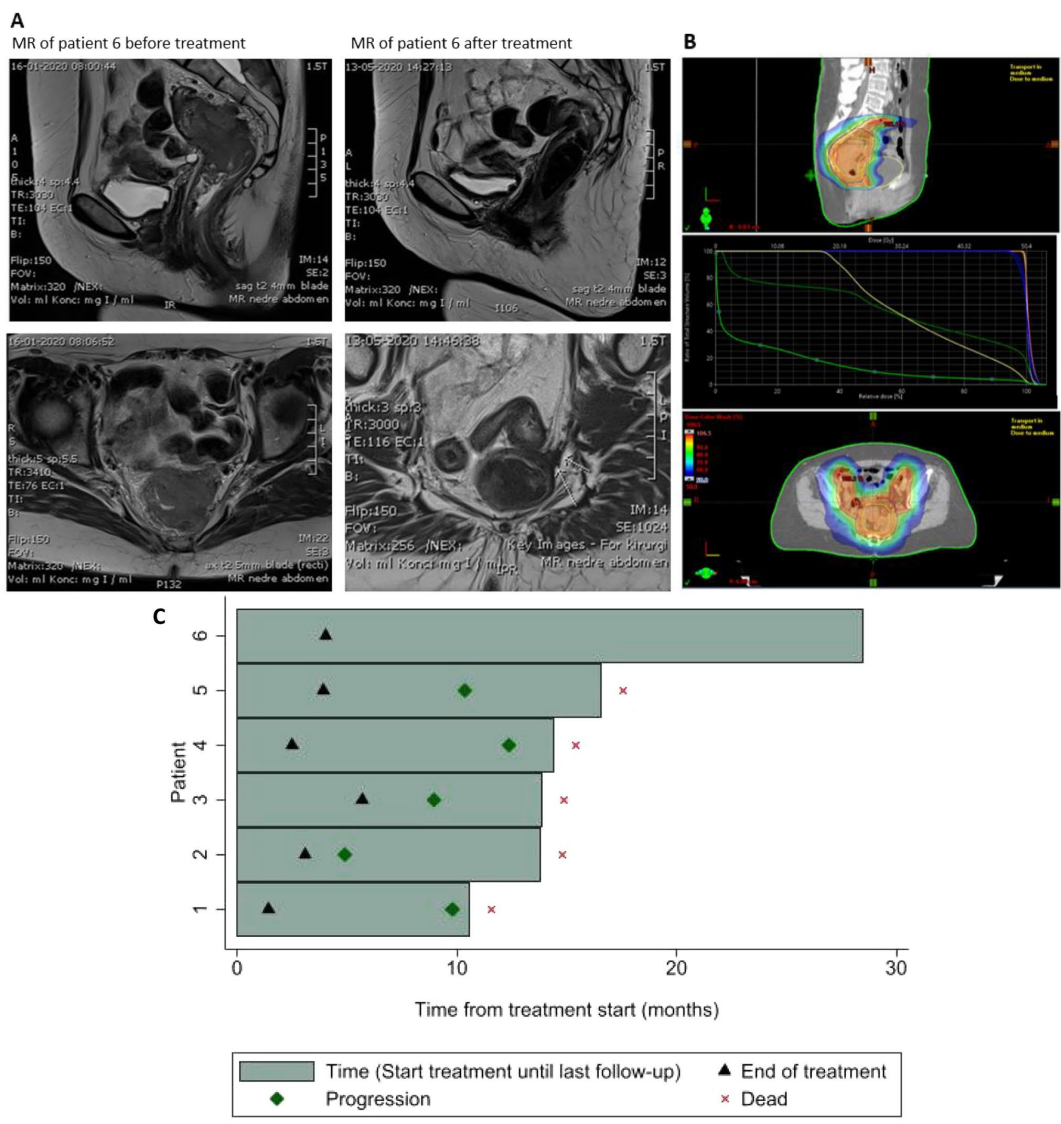
**A** MR of patient #6 before and 4 months after treatment, **B** treatment plan, **C** Swimmersplot

The patient (#6) who achieved a CR was a 63-year-old female with histologically confirmed large cell rectum PDNEC G3 with a ki-67 index of 100%. Diagnostic imaging and colonoscopy revealed a primary rectal tumour 8.6 cm from the anal verge. The tumour perforated the muscularis propria, involved the mesorectal fascia, and a vein invasion is seen that extends onto the left pelvic wall. Furthermore, the tumour perforated the visceral peritoneum by invading the loop of the sigmoideum. There were several malignant lymph nodes in the presacral mesorectal adipose tissue. Additionally, an 18F-FDG/PET with chest–abdomen–pelvic CT scan showed an advanced disease with three metastases in the liver at segments 5, 6 and 7. This led to the clinical stage T4N2M1. In an attempt to downstage the tumour, CRT was proposed for a respectability reassessment at a later date. The patient underwent one cycle of carboplatin/etoposide chemotherapy followed by concurrent CRT with a long-course (50.4 Gy/25F) regimen. The treatment was well tolerated. The RT dosimetric plan, patient baseline and post-treatment pelvic MRI are illustrated in Fig. 1. The patient underwent a successful liver metastasectomy followed by an open total mesorectal excision (TME) with temporary ileostomy and a dissection of the pelvic wall. The final pathology report showed a complete pathological response (pCR). The loop ileostomy was reversed 10 months later. After 2 years of follow-up, the patient had no evidence of the disease.

The authors affirm that patient (#6) provided informed consent for publication of the images and the case.

## Discussion

PDNEC of the rectum and anus is a very rare disease with aggressive biological behaviour, short-termed responses to therapy and inferior prognosis compared with common subtypes of rectum cancer (Nagtegaal et al. 2019; Smith et al. 2014; Chen et al. 2021). The incidence of NEN notable with rectal origin increased in recent years (Dasari et al. 2018). However, there is currently a lack of prospective studies with adequate sample sizes, which may allow for standardised disease management. Until recently, given the high propensity for metastatic spread of the anorectal PDNEC, local treatments in metastatic stages were traditionally not recommended (Janson et al. 2021; Garcia-Carbonero et al. 2016). In the adopted SCLC guidelines, platin-based chemotherapies show high response rates in PDNEC. Nevertheless, the duration of response is usually short, while the median OS is approximately 8–15 months (Shafqat et al. 2015; Sorbye et al. 2013; Fields et al. 2019).

In this case series, we focused on the role of CRT and the feasibility of liver-directed treatments as part of the multimodal approach in both locally advanced and metastatic anorectal PDNEC. As in previously published studies, our observations indicated that the CRT was well tolerated regardless of the doses of RT. Thus, Voong et al. (2017) reported that the delivered RT doses in anorectal PDNEC ranged from 45 to 60 Gy without any grade 3–4 acute toxicity. In all cases, we used the modern VMAT delivery technique which all included an RT treatment plan with conform homogeneous PTV coverage and diminishes high doses for OARs. None of our patients missed RT sessions owing to adverse events. The small intestine is commonly the main dose-limiting organ of rectal adenocarcinoma RT. A severe postoperative intestinal obstruction or intestinal adhesion has been reported in some early trials where large, small intestine volumes received high doses of radiation primarily due to suboptimal techniques (Jabbour et al. 2012). Moreover, the short treatment time of the VMAT technique and the appliance of adaptive RT strategies with daily CBTC monitoring makes it possible to perform a recalculation and reduce PTV margins. Thus, an unwanted exposure to the small intestine can be significantly reduced using the VMAT compared to other delivery techniques. In our study no patients experienced either pre- or postoperative ileus.

Due to well-recognised high response rates in PDNEC, the combination of cisplatin or carboplatin with etoposide was our first therapeutic choice in all patients. This corresponds to most currently known CRT studies in non-metastatic anorectal PDNEC (Brieau et al. 2015). In our study, all patients present with locally advanced or metastatic disease. To reduce the risk of further spread, downsize the primary tumour and improve the outcome, all patients received one cycle of induction chemotherapy followed by concurrent CRT. The benefit of this strategy compared with other CRT schedules in anorectal PDNEC still needs to be discovered. However, most of our patients experienced rapid symptom relief conditioned by the primary tumour, such as pain, tenesmus and rectal bleeding after an initial chemotherapy cycle.

The application of CRT in the treatment of non-metastatic anorectal PDNEC has been growing in the last decade. High disease control rates of up to 93% in this group of patients were observed by several investigators when CRT was used as definitive treatment (Ueberroth et al. 2021; Voong et al. 2017; Brieau et al. 2015). This correlated with our observations in a radiological confirmed LRTC at the disease’s progression time in four patients.

Surgery in localised anorectal PDNEC is still under debate. A recently published SEER database study has shown that non-surgical approaches had an adverse prognostic influence on OS in rectal NEC (Shi et al. 2020). However, a retrospective study of 126 patients with high-grade NEC of the colon and rectum outlined that surgery was not associated with a significant improvement of OS compared to CRT without primary tumour resection (Smith et al. 2014). Later, Brieau et al. showed similar results in 24 patients with anorectal PDNEC (Brieau et al. 2015). Furthermore, a SEER database retrospective study of 71 patients with small cell rectum NEC demonstrated a significantly longer 1-year survival and median OS in patients treated conservatively with RT alone compared to patients who underwent tumour resection (Modrek et al. 2015).

Similarly, one elderly patient from our cohort with locally advanced rectal MiNEN and a history of multiple comorbidities experienced a stable disease over a 12-month follow-up after CRT.

Overall, there is broad agreement among experts that perioperative chemotherapy or CRT may improve outcomes in non-metastatic anorectal PDNEC (Smith et al. 2014; Pommergaard et al. 2021; Pellat et al. 2019). However, local treatments for limited metastatic disease in rectal adenocarcinomas and anal squamous cell carcinomas, associated with a survival benefit, remain the primary treatment choice in metastatic anorectal PDNEC, the systemic chemotherapy (Janson et al. 2021; Joharatnam-Hogan et al. 2020). In our study, three patients were presented with synchronous liver metastases. An adequate clinical response assessment to CRT and consequent discussions in MDT meetings were performed for each case. Two patients were offered RFA of liver metastases followed by the surgical resection of the primary tumour. One patient was found suitable for hepatic resection before TME. This patient achieved a pCR in both primary and metastatic resected specimens and had not relapsed (28 months after surgery). Comparatively, pCR after neoadjuvant or definitive CRT was previously observed in individual patients with anorectal PDNEC (Smith et al. 2014). However, the distant recurrence was predominant and occurred in about 90% of patients regardless of operative or non-operative treatments (Smith et al. 2014).

PDNEC is a morphologically heterogeneous group of malignancies. In our cohorts, no remarkable differences in clinical behaviour were perceived in pure anorectal NEC compared to MiNEN variants (Laenkholm et al. 2021). Although the survival benefit of neoadjuvant CRT has not yet been confirmed, it remains a standard treatment for LARC adenocarcinomas (Joharatnam-Hogan et al. 2020). Recently published meta-analyses based on six studies, including in total 12,812 patients, demonstrated that while the rate of distant metastases was lower in the neoadjuvant chemotherapy group, neoadjuvant CRT led to a higher rate of primary tumour downstaging/downsizing, a pCR as well as a higher R0 resection rate (Lin et al. 2021). Due to a well-recognised aggressive biological behaviour and a dismal prognosis, chemotherapy remains the treatment of choice in advanced stages of anorectal PDNEC (Sorbye et al. 2023). Prospective studies comparing neoadjuvant CRT with perioperative chemotherapy without RT in LA anorectal PDNEC are missing. Recently, it was shown that additional preoperative, postoperative or perioperative chemotherapy administrations were associated with improved rates of survival in patients with digestive PDNEC (Fields et al. 2019; Alese et al. 2019; Dasari et al. 2022). However, the reported percentage (about 30%) of margin-positive resections after surgery remains relatively high (Alese et al. 2019). In our study, all patients had cT3–cT4 tumours at diagnosis. Radical surgery was possible in four patients due to a significant decrease in primary tumour size. Based on reviewed postoperative pathological results, we observed a pCR on the T-site (ypT0) in two and ypR0 resection margins in three out of four operated patients. Furthermore, evidence of locoregional failure at disease progression was not observed in four patients. This result suggests that CRT may improve the chances of primary tumour downstaging/downsizing for a microradical resection in anorectal PDNEC and reduce risk of locoregional recurrence.

Up to 80% of PDNEC patients have synchronous liver metastases at the time of diagnosis (Nagtegaal et al. 2019; Janson et al. 2021). Studies regarding the role of multimodal approaches, including various liver-directed treatments in metastatic anorectal PDNEC, are tremendously sparse. Patient characteristics and tumour histopathological features are poorly described in these reports. A prior study which included 32 patients from two Nordic gastroenteropancreatic neuroendocrine carcinoma (GEP-NEC) registries showed a prolonged median OS of 35.9 months following resection of liver metastases (Galleberg et al. 2017). Four patients, including one with rectal NEC, were recurrence free 60–184 months after the surgery. All patients had PDNEC of non-small cell morphology with Ki67 of about 70% and few liver metastases with unilateral presentation. Three patients received either neoadjuvant or adjuvant chemotherapy. However, no specific patient selection factors were found which could help explain these promising results. Nevertheless, the role of chemotherapy in long-term PFS in this group of patients is uncertain.

Anorectal PDNEC of small cell morphology has a worse prognosis in about 70% of patients with distant metastases at diagnosis (Dasari et al. 2022). In our study, one patient (#1) had small cell rectum PDNEC with solitary synchronous liver metastasis, which was successfully treated with RFA before CRT. Although the patient experienced a pCR in the primary tumour site and the ypR0 resection was reported, an early distant intrahepatic recurrence was detected. This observation corresponded to a previously published meta-analysis based on 190 patient cases of small cell rectal PDNEC (Qasem et al. 2016). This study indicated that combination surgery with chemotherapy and RT could be offered to patients with limited disease, being seemingly superior to dual modalities. Systemic chemotherapy without radiation therapy should be reserved for palliative treatment of metastatic disease, due to the poor prognosis. These findings correspond to our data, reinforcing the earlier initiation of systemic therapy in PDNEC. Furthermore, the often-observed treatment-associated decrease of the ki67-index after CRT did not influence the course of the disease. The prognostic significance of this phenomenon in PDNEC needs further evaluation (Vyas et al. 2021).

Our observations suggest that an accurate selection of patients with anorectal PDNEC with the best chance of achieving a long-term outcome following multimodal treatment is needed. Nevertheless**,** an appropriate assessment for such selection still needs to be included. Despite the high specificity of currently available neuroendocrine biomarkers, the sensitivity remains low (Dam et al. 2020). In recent years, cancer treatment strategies have become more personalised and increasingly incorporated into treatment decisions. Emerging studies show a promising performance of cell-free DNA analysis for detecting microscopic residual disease after CRT (Callesen et al. 2022). Thus, an increased risk of recurrence, shorter time to relapse and shorter disease-free survival in patients with locally advanced rectum adenocarcinomas treated with CRT showed a correlation with high baseline plasma level cell-free DNA (Callesen et al. 2022). We suggest that this concept be transferred to an appropriate selection of patients with anorectal PDNEC for multimodal treatment.

The main limitation of this study is the small number of cases consisting primarily of retrospective observations as well as the heterogeneity of the study population. Nevertheless, a single specialised centre had consistently provided disease staging as well as multidisciplinary decision-making of the disease management in all cases. This study showed that most patients experienced a primary tumour shrinkage which allowed for radical surgery following the initial CRT, potentially improving the chances for long-term survival as a result.

## Conclusion

In this case series, we focused on the role of CRT and the feasibility of liver-directed treatments as part of the multimodal approach in both locally advanced and metastatic anorectal PDNEC. The treatment was well tolerated and indicated a grade of local control by confirmed LRTC at the time of progression in four patients. One patient even achieved a complete response. Our findings support other studies that suggest that CRT gives a high grade of local disease control. The role of surgery in these neoplasms remains controversial.

In the future, an accurate selection of patients with anorectal PDNEC with the best chance of achieving a long-term outcome following multimodal treatment is needed. Furthermore, all PDNEC of the rectum and anus patients should be prioritised to be included in clinical trials to generate more substantial evidence for managing their disease.

## Data Availability

The datasets generated during and analysed during the current study are available from the corresponding author on reasonable request.
